# A novel *COL4A5* splicing mutation causes alport syndrome in a Chinese family

**DOI:** 10.1186/s12920-024-01878-8

**Published:** 2024-04-26

**Authors:** Suyun Chen, Guangbiao Xu, Zhixin Zhao, Juping Du, Bo Shen, Chunping Li

**Affiliations:** 1 Department of Clinical Laboratory, Taizhou Hospital of Zhejiang Province Affiliated to Wenzhou Medical University, Linhai, China; 2 Key Laboratory of System Medicine and Precision Diagnosis and Treatment of Taizhou, Linhai, China; 3 Department of Nephrology, Taizhou Hospital of Zhejiang Province Affiliated to Wenzhou Medical University, Linhai, China; 4 Department of Neurosurgery, Taizhou Hospital of Zhejiang Province Affiliated to Wenzhou Medical University, Linhai, China

**Keywords:** Alport syndrome, *COL4A5* gene, Minigene assay, Aberrant splicing

## Abstract

**Background:**

Alport syndrome (AS) is characterised by haematuria, proteinuria, a gradual decline in kidney function, hearing loss, and eye abnormalities. The disease is caused by mutations in *COL4An* (*n* = 3, 4, 5) that encodes 3–5 chains of type IV collagen in the glomerular basement membrane. AS has three genetic models: X-linked, autosomal recessive, and autosomal dominant. The most common type of AS is X-linked AS, which is caused by *COL4A5*.

**Methods:**

We enrolled children with renal insufficiency and a family history of kidney disorders. The proband was identified using whole-exome sequencing. Sanger sequencing was performed to verify the mutation site. Minigene technology was used to analyse the influence of mutant genes on pre-mRNA shearing, and the Iterative Threading ASSEmbly Refinement (I-TASSER) server was used to analyse the protein structure changes.

**Results:**

The proband, together with her mother and younger brother, displayed microscopic haematuria and proteinuria, Pathological examination revealed mesangial hyperplasia and sclerosis. A novel mutation (NM_000495.5 c.4298-8G > A) in the intron of the *COL4A5* gene in the proband was discovered, which was also present in the proband’s mother, brother, and grandmother. In vitro minigene expression experiments verified that the c.4298-8G > A mutation caused abnormal splicing, leading to the retention of six base pairs at the end of intron 46. The I-TASSER software predicted that the mutation affected the hydrogen-bonding structure of COL4A5 and the electrostatic potential on the surface of the protein molecules.

**Conclusions:**

Based on the patient’s clinical history and genetic traits, we conclude that the mutation at the splicing site c.4298-8G > A of the *COL4A5* gene is highly probable to be the underlying cause within this particular family. This discovery expands the genetic spectrum and deepens our understanding of the molecular mechanisms underlying AS.

**Supplementary Information:**

The online version contains supplementary material available at 10.1186/s12920-024-01878-8.

## Introduction

Alport syndrome (AS) is an inherited kidney disease characterised by haematuria, proteinuria, and gradual decline in kidney function. It is often associated with hearing loss and eye abnormalities [1]. AS arises from abnormalities in type IV collagen, a crucial building block of the basement membrane (BM) found in the kidney, eye, and cochlea. AS is caused by mutations in the genes *COL4A3*, *COL4A4*, and *COL4A5*, encoding the chains 𝛼3, 𝛼4, and 𝛼5 of type IV collagen, respectively [2]. AS is estimated to affect approximately 1 in every 5,000 to 1 in every 53,000 individuals in different populations [3]. The occurrence of AS in children who undergo renal biopsy varies between 1% and 12%, depending on the specific indications for the biopsy [4–6].

AS has three different inheritance patterns: X-linked Alport syndrome (XLAS), autosomal recessive, and autosomal dominant [7]. XLAS, caused by mutations in the *COL4A5* gene, is the predominant form of AS, accounting for approximately 85% of the cases [8]. The Xq22 chromosome is where the *COL4A5* gene is situated [9]. Currently, the Human Gene Mutation Database (http://www.hgmd.org) contains over 1000 different versions of the *COL4A5* gene. Approximately 60% of pathogenic mutations in XLAS are missense mutations, whereas frameshift and nonsense mutations account for 20% and 10%, respectively. The remaining 10% are attributed to canonical splicing mutations [10]. Although the exact correlation between genotypes and phenotypes in XLAS is not fully understood, it is commonly accepted that the severity of phenotypes is less pronounced with missense mutations than with truncated mutations [11].

Patients with splicing abnormalities often exhibit severe illness [12]. In this study, we used whole-exome and Sanger sequencing techniques to discover novel splicing variations in a Chinese family experiencing haematuria and proteinuria. Next, we conducted an additional analysis to examine the impact of the genetic mutation on transcription using a minigene assay.

## Materials and methods

### Subjects

**Fig. 1:**
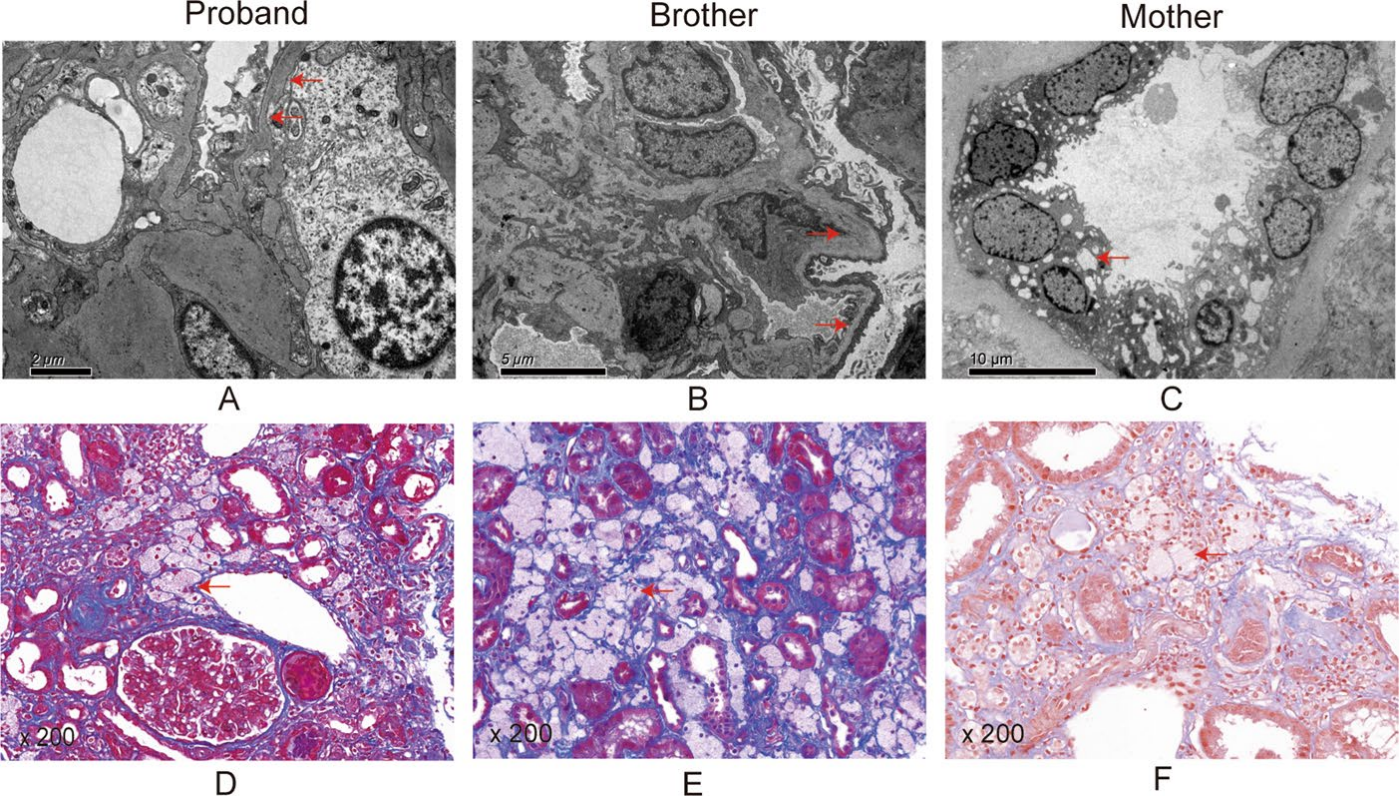
Results of renal biopsy. (**A**, **B**) Electron microscopy shows irregular thickening and thinning of glomerular basement membrane. (**C**) Electron microscopy shows vacuolar degeneration in the renal tubular epithelium. (**D**, **E**, **F**) Masson staining shows infiltration of foamy cells in the interstitial

On 1 January 2022 a 14-year-old boy, identified as the proband (III-1), was admitted to our hospital’s nephrology department because of renal dysfunction. The proband presented with haematuria and proteinuria. The results of the auxiliary examination were as follow (Table 1): serum urea, 14.21 mmol/L; serum creatinine (Scr), 259 µmol/L; 24-hour urinary protein, 5175 mg; estimated glomerular filtration rate (eGFR), 31 mL/min/1.73 m^2^; urine protein to creatinine ratio (uPCR), 9.66 g/g; and haemoglobin level 115 g/L. The patient had a family history of kidney disease. His maternal uncles (II-1 and II-2) died due to renal disease when they were 24 and 10 years old, respectively. His mother had chronic nephritis. During the examination, the proband exhibited myopia, wheres the auditory test revealed no signs of hearing loss. The renal parenchyma exhibited heightened echogenicity as observed on Doppler ultrasonography of the urinary system. Renal biopsy under light microscopy showed focal and segmental glomerulosclerosis (FSGS), with spherical sclerosis accounting for 59%, and many foam cells appearing in the renal interstitium (Fig. 1D). The renal electron microscopy showed diffuse fusion of glomerular foot processes, while the BM exhibited varying thickness (100–690 nm) as observed through electron microscopy (Fig. 1A). Based on the results of microscopic haematuria and proteinuria, family history of nephropathy, renal pathology and electron microscopy, the physicians strongly suspected AS [13].

**Table 1:** Clinical characteristics and genotypes of individuals from a Chinese Family with *COL4A5* mutation

	Proband	Brother	Mother	grandmother	grandfather
COL4A5 Mutation Position	c.4298-8G > A (hemi)	c.4298-8G > A (hemi)	c.4298-8G > A (Het)	c.4298-8G > A(Het)	-
Sex	M	M	F	F	M
Age (yr)	13	11	31	72	76
Blood pressure (mmHg)	114/60	120/89	171/130	146/74	182/93
Occult blood	2+	3+	±	±	-
Red blood cells under microscope (0–3/HP)	2+	3+	5–10	-	-
Proteinuria	2+	4+	2+	2+	1+
Creatinine (59–104) µmol/L	259	103	205	76	128
urea (3.1-8) mmol/L	14.21	7.84	10.24	4.86	7.64
Uric acid (208–418) µmol/L	263	315	459	300	472
24 h urinary protein (< 150 mg)	5175	2838	560	ND	ND
uPCR (< 0.2) g/g	9.66	6.76	1.71	0.76	0.44
eGFR (90–120) mL/min/1.73 m²	31	96	27	68	35
Renal ultrasound	Enhanced echo of renal parenchyma	Enhanced echo of renal parenchyma	Enhanced echo of renal parenchyma	ND	ND
Renal pathology	FSGS(Glomerular sclerosis accounts for 59%)	FSGS(Glomerular sclerosis accounts for 32%)	proliferative sclerosing glomerulonephritis(Glomerular sclerosis accounts for 50%)	ND	ND
Ocular lesions	200 degree myopia	200 degree myopia	1000 degree myopia	N	myopia
Hearing loss	**-**	**-**	**-**	**-**	**-**

Hemi: hemizygote; Het: heterozygote; F: female; M: male; ND: not done; ±: weakly positive; -: negative; uPCR: urine protein to creatinine ratio; eGFR: estimated glomerular filtration rate; FSGS: focal segmental glomerulosclerosis

The mother (II-4) was admitted to the hospital in October 2020 with kidney disease. Auxiliary examination has the following results: urine protein, 2+; Scr, 205 umol/l; uric acid, 459 umol/L; urine protein, 2+; eGFR, 27 mL/min/1.73m^2^; and uPCR, 1.71 g/g. She underwent a renal biopsy, which revealed many foam cells appearing in the renal interstitium through Masson staining (Fig. 1F) and vacuolar degeneration in the renal tubular epithelium through electron microscopy (Fig. 1C). She had 1000 degree myopia and a history of hypertension.

The 11-year-old younger brother (III-2) experienced microscopic haematuria and proteinuria in February 2022  , with eGFR of 96 mL/min/1.73 m^2^ and uPCR of 6.76 g/g. Additionally, he had myopia of 200 degrees. Renal biopsy under light microscopy showed FSGS, with spherical sclerosis accounting for 32%, and many foam cells in the renal interstitial (Fig. 1E), similar to the proband. Electron microscopy revealed varying thicknesses of the BM ranging from 200 to 650 nm (Fig. 1B). A few BMs exhibit minor layering and local tearing. He was also strongly suspected of AS.

His healthy father (II-3) declined to undergo examination. The proband’s grandmother (I-2) is generally healthy, with eGFR of 68 mL/min/1.73m^2^ and uPCR of 0.76 g/g. The proband’s grandfather(I-1) experienced hypertension, and went to the hospital 10 years ago due to kidney disease, with eGFR of 35 mL/min/1.73m^2^ and uPCR of 0.44 g/g. He also underwent Chinese Herb Medicines treatment (specific details unknown) for six months after discharge, later his physical health remained in good condition.

### Genetic testing

Genomic DNA was extracted from the proband and his family using a Blood Genomic DNA Extraction Kit (QIAGEN, Germany). The IDT xGen Exome Research Panel vl.0 (Integrated DNA Technologies, Inc., United States) was used to construct a DNA library. The Illumina NovaSeq 6000 Sequencing System was used to perform whole-exome sequencing (WES) by next-generation sequencing (NGS), generating 150 bp paired-end sequences. The DNA sequence was matched with the human reference sequence UCSC hg19/GRCh37. and alignment analysis was performed using BWA software. Genes related to glomerular disease, nephritis, kidney disease, and urinary system are focused on and analyzed (Additional file 1: Table S1). The variation annotation databases included the NCBI Reference Sequence Database (RfeSeq), Single Nucleotide Polymorphism Database (dbSNP), 1000 Genomes Project, and Genome Aggregation Database (gnomAD). The pathogenicity of the variants was annotated using the ClinVar database, the Human Gene Mutation Database and Online Mendelian Inheritance in Man (OMIM), and standards and guidelines for the Interpretation of Sequence Variants of American College of Medical Genetics and Genomics (ACMG).

To verify the variant, the proband and his family members, including his mother, younger brother, and grandparents, were confirmed by Sanger sequencing using an ABI 3500Dx sequencer (Applied Biosystems, Inc.), following the manufacturer’s guidelines. The following primer sequences were used: COL4A5 forward: (5′-AGCCCATGATATCTGACAATGC-3′) and reverse (5′-GTTTTCAATTCGGAAGCCCC-3′). Hangzhou Dian Medical Laboratory Company of China commercially completed the task.

### In silico analysis

To predict the potential impact of mutations in introns, the bioinformatic splicing tools Human Splicing Finder (HSF) version 3.0 1(http://www.umd.be/HSF3/HSF.shtml) and SpliceAI (https://spliceailookup.broadinstitute.org/) were used.

### Construction of recombinant plasmids

For nested PCR, two sets of primers were created using the genomic DNA of normal individual as a template: round 1 of nested primers 245,465-COL4A5-F and 248,738- COL4A5-R (the length of PCR products is 3274 bp) and round 2 of nested primers 245,699-COL4A5-F and 248,432-COL4A5-R (the length of PCR products is 2734 bp) (Table 2). The diagram of primers positions for specific alleles is provided in Additional file 2: Fig S1.

**Table 2:** Primers used in the study

Name	Primers	Sequence (5′-3′)	Product length
Round 1 amplification primers for Nested PCR	245,465-COL4A5-F	ctaactggtgtctagctggc	3274 bp
	248,738-COL4A5-R	gaatggcccagacaatgaga	
Round 2 amplification primers for Nested PCR	245,699-COL4A5-F	catcccagaagcaaatggat	2734 bp
	248,432-COL4A5-R	aggctgaggcagaagaattg	
pcMINI-N-wt/mut full-length amplification	pcMINI-N-COL4A5-HindIII-F	acttaagcttatgggtccaactggccctccagga	2119 bp
	pcMINI-N-COL4A5-ECORI-R	tgcagaattctttgatctatgaaccaagat	
pcMINI-N mut left half fragment amplification	pcMINI-N-COL4A5-HindIII-F	acttaagcttatgggtccaactggccctccagga	1473 bp
	COL4A5-mut-R	cgggtacctatttctacgaaataatcagta	
pcMINI-N mut right half fragment amplification	COL4A5-mut-F	tactgattatttcgtagaaataggtacccg	662 bp
	pcMINI-N-COL4A5-ECORI-R	tgcagaattctttgatctatgaaccaagat	

Using the product of the second round of nested PCR as a template and pcMINI-N-COL4A5 HindIII-F and pcMINI-N-COL4A5 EcoRI-R as primers, a fragment 2119 bp of pcMINI-N-COL4A5-wt was amplified. The pcMINI-N vector is provided in Additional file 3: Fig S2.

Using the product of the second round of nested PCR as a template, with COL4A5 mut-R and pcMINI-N-COL4A5 Hind III-F as primers, the left half fragment of pcMINI-N-COL4A5 mut was amplified to 1473 bp. Using COL4A5 mut-F and pcMINI-N-COL4A5 EcoRI-R as primers, the right half fragment of pcMINI-N-COL4A5 mut was amplified to obtain 662 bp. Using a 1:1 mixture of left and right segments as a template, with pcMINI-N-COL4A5 Hind III-F and pcMINI-N-COL4A5 EcoRI-R as primers, a 2119-bp fragment of pcMINI-N-COL4A5 mut was amplified. Using three-step PCR cycling with a melting temperature (Tm) of 57 °C for 30 s per cycle, for 30 cycles in total.

**Fig. 2:**
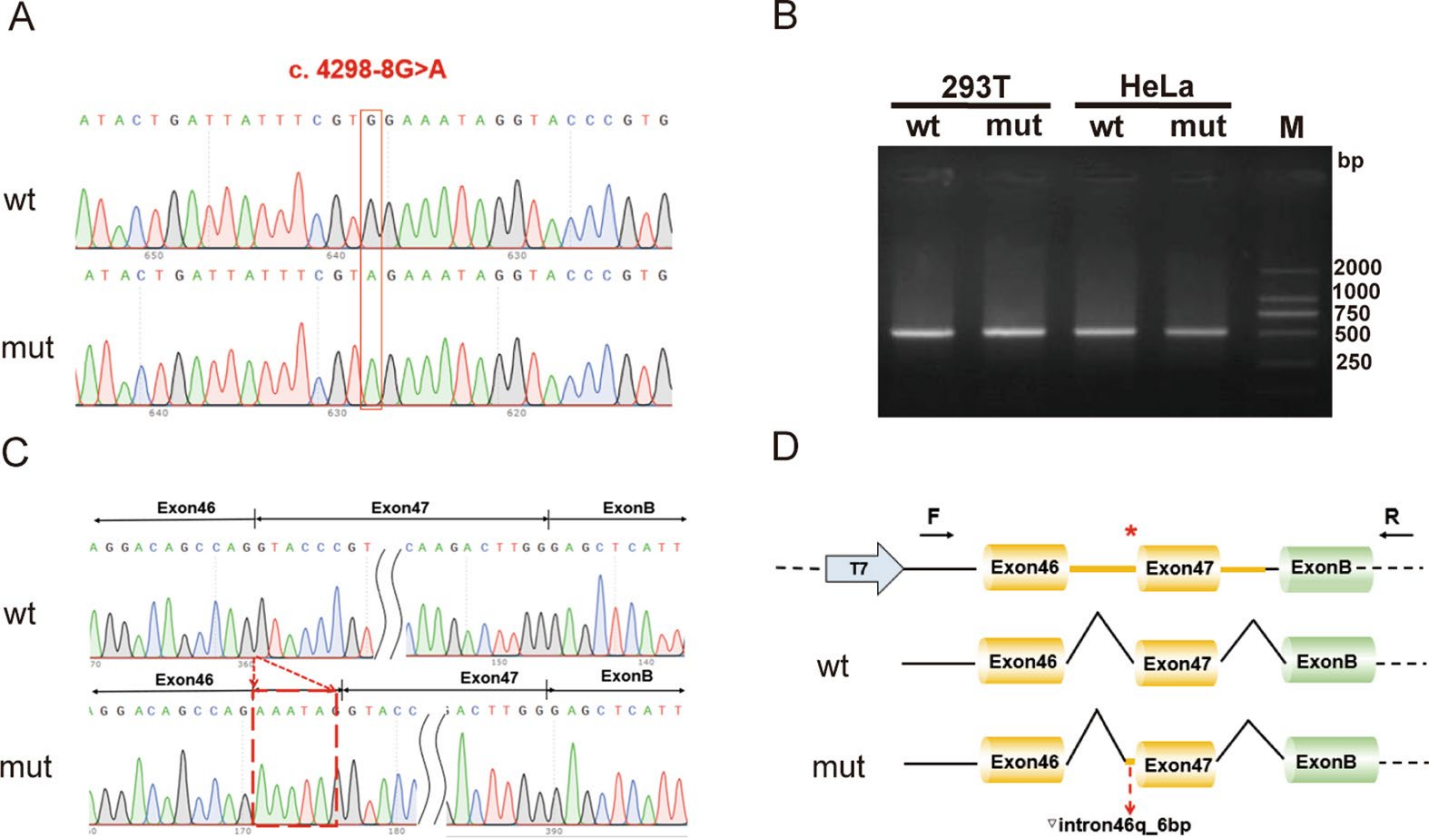
Results of the mini-gene splicing assay. (**A**) Sanger sequence of minigene in wt and mut group (c. 4298-8 G > A). (**B**) The agarose gel electrophoresis of RT-PCR fragments showed that the product length was similar between wt and mut groups. (**C**) The Sanger sequences showed a 6 bp base (AAATAG) retention at the right of intron 46 in the variant group. (**D**) The schematic diagram showed the minigene comprising exon 46, exon 47, and intron 46 of the wt or mut (c. 4298-8 G > A). The variant c. 4298-8 G > A affected the normal splicing of *COL4A5* mRNA resulting in 6 bp base retention at the right of intron 46. “*” represents variant location

The PCR products were digested using the HindIII and EcoRI restriction enzymes. Subsequently, the digested products were ligated into identical restriction sites of the pcMINI-N vector (Wuhan Bioegle Biological Technology and Science Co., Ltd., Wuhan, China) to generate recombinant pcMINI-N-COL4A5-wt/mut. Recombinant plasmids were digested with HindIII and EcoRI, and verified by gene sequencing (Fig. 2A).

### Transfection of eukaryotic cells

The pcMINI-N-COL4A5-wt and pcMINI-N-COL4A5-mut vectors were transfected into HEK-293T and HeLa cells using a Liposomal Transfection Reagent. The transfected cells were then cultured for 48 h hours prior to analysis.

### Minigene transcription analysis

RNA was isolated from 293T and HeLa cells using the TRIzol technique, followed by reverse transcription of cDNA using a reverse transcription kit (Thermo) according to the manufacturer’s guidelines. RT-PCR amplification was performed using the designed primers, followed by detection of the PCR products through using agarose gel electrophoresis and Sanger sequencing.

### The evolutionary conservation analysis of amino acid residues and the 3D models structural analysis of mutant proteins

The evolutionary conservation of the COL4A5 candidate variant was analysed by protein sequence alignment of various species using the Jalview software. The wild-type amino acid sequence and cDNA sequence of the *COL4A5* gene were obtained from UCSC (http://genome.ucsc.edu/), and the mutant amino acid sequence of COL4A5 was obtained by marking the mutation on the cDNA using SnapGene software. Then, the 1500 amino acids from 1 to 1500 of the wild-type and mutant amino acid sequences in COL4A5 were modelled using the I-TASSER server to evaluate the influence of the variable region, and the 3D structure of the protein was visualized using PyMol software.

## Results

### Genetic findings

**Fig. 3:**
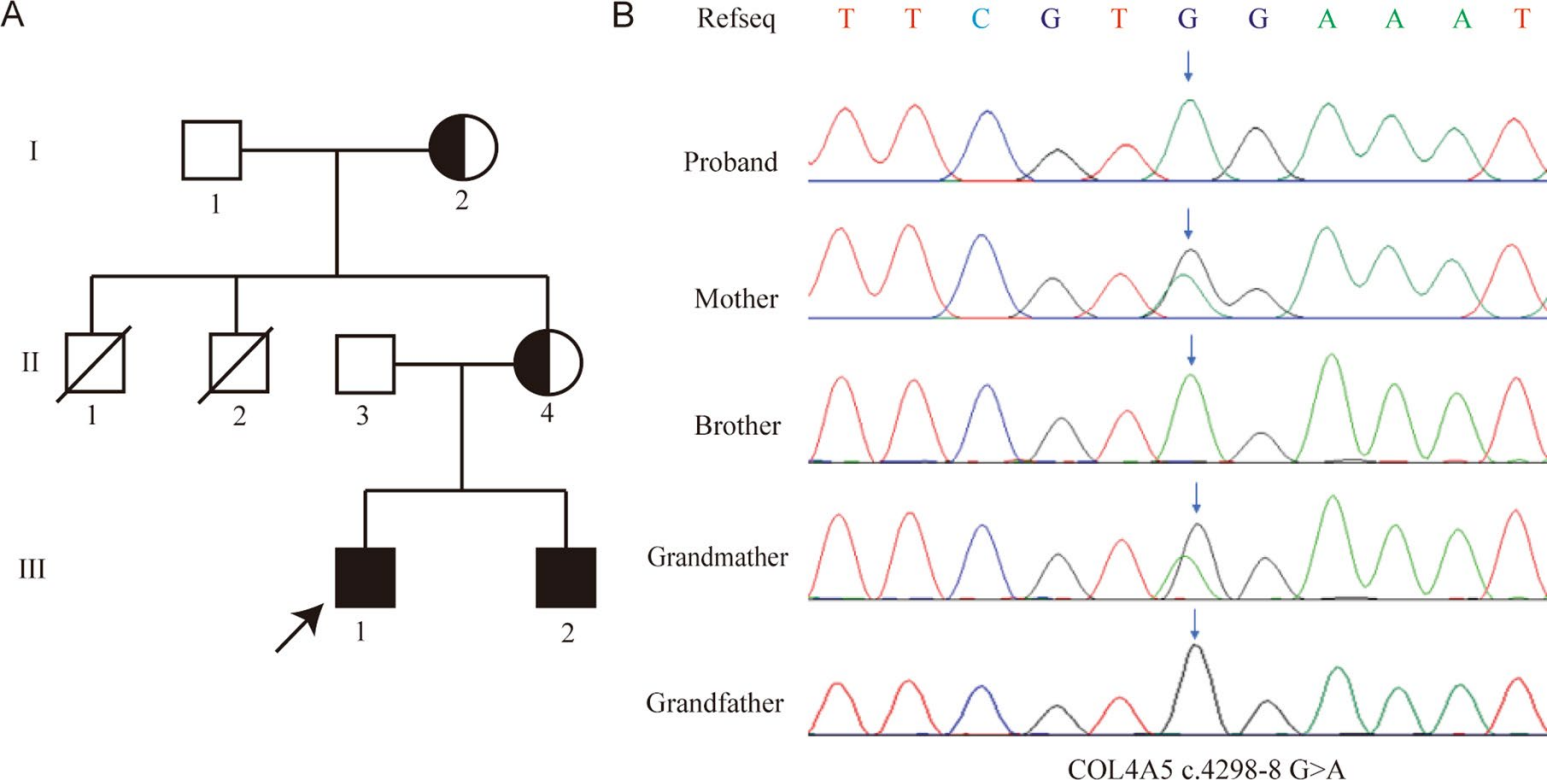
Family pedigree and confirmation of the variant of COL4A5. (**A**) The pedigree of the family. Filled symbols represent affected individuals (black: the 4298-8 G > A variant carriers). Arrow indicates the proband. (**B**) Identification of *COLAA5* variant in the family through Sanger sequencing, the arrows represent sites of variant

The pedigree of the family is presented in Fig. 3A. Whole-exome sequencing (WES) analysis was conducted on the proband. WES revealed a novel mutation (c.4298-8G > A) in the intron of the *COL4A5* gene (NM_000495.5), located on the X chromosome. Sanger sequencing was used to test the DNA samples from the mother, brother, and grandparents to confirm the variant. The test revealed that the younger brother carried the c.4298-8G > A mutation in a hemizygous state, whereas the proband’s mother and grandmother carried this mutation in a heterozygous state. However, a *COL4A5* mutation was not detected in the proband’s grandfather (Fig. 3B). The mutation c.4298-8G > A in the *COL4A5* gene has not been previously documented in the gnomAD database.

### Splice effect of the *COL4A5* c. 4298-8G > A variant

According to the HSF software, it is probable that the intronic variant c. 4298-8G > A affects mRNA splicing. According to the Splice AI software, the acceptor splicing site exhibited a substantial alteration in splicing, with a range of 0.75 to 0.9 before and after the mutation. To demonstrate the impact of the c. 4298-8G > A mutation on mRNA splicing, we performed a minigene splicing experiment. PCR amplification was used to analyse the minigene splicing products using plasmid-specific primers, and visualisation was achieved using polyacrylamide gel electrophoresis. The electrophoretic outcomes of the wild-type and c. 4298-8G > A transfections demonstrated that the mutant band resembled that of the wild-type (Fig. 2B and Additional file 4: Fig S3). Sanger sequencing-verified amplicons in the variant group exhibited retention of a 6-bp base (AAATAG) to the right of intron 46 (Fig. 2C), which aligns with the *in silico* analysis outcome. The cDNA and protein representation were approximately 4297_4298ins aaatag p.Pro1432_Gly1433insGluIle. The schematic diagram of minigene construction and the c. 4298-8 G > A mutation revealed abnormal splicing (Fig. 2D).

### Analysis of the influence of mutation on protein structure by model

**Fig. 4:**
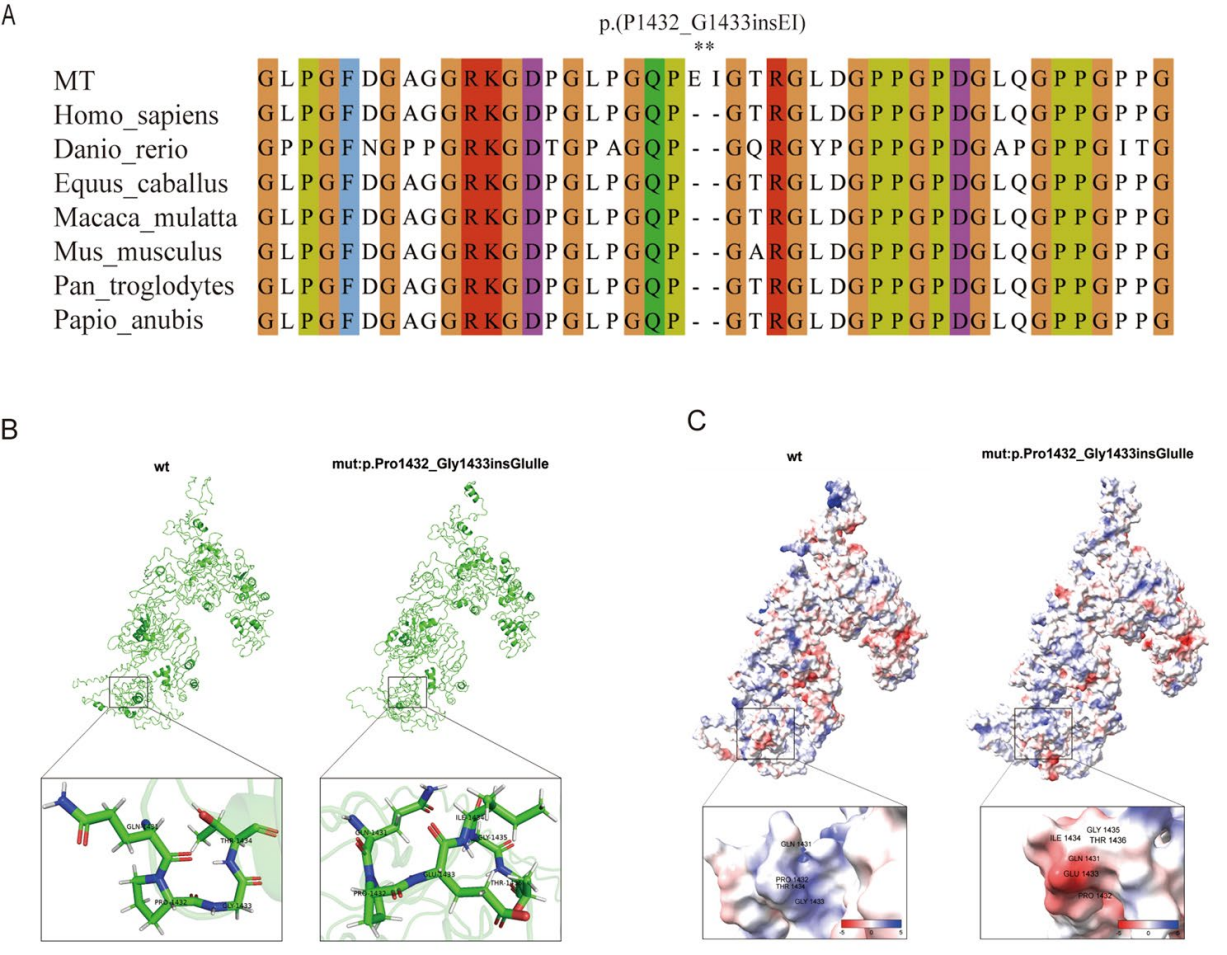
Analysis of COL4A5 mutation. (**A**) Evolutionary conservation of amino acid residues altered by p.(P1432_G1433insEI) across different species. NCBI accession numbers are Homo sapiens: NP_000486.1; Danio rerio: XP_021333076.1; Equus caballus: XP_005614463.1; Macaca mulatta: XP_014983488.2; Mus musculus: XP_006528759.1; Pan troglodytes: XP_016798385.2; Papio Anubis: XP_031516835.1. Asterisk (*) means Inserted amino acids. (**B**) Prediction of the change in the three-dimensional (3D) structure of hydrogen bond by COL4A5 p.( P1432_G1433insEI) variant. (**C**) Prediction of the change in the three-dimensional (3D) structure of surface electrostatic potential by COL4A5 p.( P1432_G1433insEI) variant

Evolutionary conservation analysis showed that the protein insertion region was highly conserved among different species (Fig. 4A). The I- TASSER program was used to predict the arrangement of the wild-type and mutant COL4A5 proteins. The models of the three-dimensional (3D) structure of the protein revealed a modification in the hydrogen bond structure of amino acids 1432_1433(Fig. 4B). Additionally, the electrostatic potential near the 1432_1433 mutation on the surface of the protein shifted from electropositive to electronegative following the COL4A5 p.(P1432_G1433insEI) variant (Fig. 4C). This alteration could potentially affect the spatial structure of proteins.

### Pathogenicity analysis of variation

According to the ACMG/AMP guidelines [14], the *COL4A5* c.4298–8G > A variant was classified as likely pathogenic (PP4 + PP3 + PP1 + PM2Supporting + PM4Supporting). The evidence is as follows. PP4: The clinical manifestations were remarkably in line with a genetic disorder resulting from an abnormality in the *COL4A5* gene. PP3: According to the HSF and Splice AI software, the mutation has the potential to affect gene splicing. PP1: The mutation segregates with the disease phenotype within the family. PM2_Supporting: This variant is uncommon and is not listed in the gnomAD database. PM4_Supporting: The mutation in the splicing region caused the emergence of a new splicing acceptor, which led to the preservation of six base pairs on the right side of intron 46 and the addition of 2 amino acids. By combining the clinical manifestations and genetic test results, we concluded that the proband had AS caused by a novel *COL4A5* splice mutation.

## Discussion

The AS phenotype is characterised by isolated non-progressive haematuria to progressive nephropathy with extrarenal abnormalities [15]. Approximately 60% are estimated to develop end-stage renal disease (ESRD) after 40 years of age. The most prevalent indications in these patients were microhaematuria and proteinuria. Mutations in COL4A5 are the main causes of AS. Here, we report a case of AS with a novel *COL4A5* mutation, c.4298-8G > A. The proband’s age at onset was 13 years, and he exhibited microhaematuria and proteinuria with elevated levels of creatinine in the blood. The proband’s family history included two maternal uncles who passed away because of kidney disease, and a mother who had kidney disease, all of which led to a high suspicion of AS. High-throughput sequencing and analysis of approximately 20,000 gene exons and ± 10-bp intron regions in the proband’s genomic DNA revealed the *COL4A5* mutation c.4298-8G > A. The pathogenicity of the *COL4A5* variant was confirmed by clinical manifestation, human genome variation database analysis, functional software prediction, and minigene assay. According to the ACMG guidelines, the *COL4A5* variant was classified as a likely pathogenic variant.

The gene *COL4A5* is situated in region Xq22.3 and comprises a total of 51 exons. The translated product consists of 1685 amino acid residues, including a signal peptide of 26 residues, a collagenous domain of 1430 residues encoded by exons 2–47, which begins with a noncollagenous sequence of 14 residues, and a gly-Xaa-Yaa-repeat sequence interrupted at 22 locations. This is followed by a carboxyl-terminal non-collagenous domain of 229 residues encoded by exons 47–51 [16]. The main genetic pattern of AS caused by the *COL4A5* mutation is X-linked dominant inheritance. Sanger sequencing confirmed that the mutation *COL4A5* c.4298-8G > A was present in the proband’s mother, brother, and grandmother, indicating a typical X-linked inheritance pattern. Almost all male patients with XLAS have microscopic haematuria and proteinuria; the incidence of hearing loss is 32–83%, and the incidence of eye lesions is 6–35% [11, 17–19]. More than 90% of female heterozygotes with XLAS have microscopic haematuria, approximately 70% have proteinuria, 6–28% have hearing loss, and 2–15% have eye lesions [20–22]. In this study, both the proband and younger brother showed microscopic haematuria and proteinuria, as well as myopia and no hearing loss. The mother of the heterozygous carrier had microscopic haematuria, proteinuria, and high myopia, whereas the grandmother had proteinuria without any other abnormal manifestations. In XLAS, hemizygous male patients usually exhibit more severe characteristics that advance to ESRD during early or middle adulthood than the heterozygous female [17, 20]. In this case, the proband’s two uncles passed away from ESRD when they were 24 and 10 years old, respectively. At the ages of 14 and 12 years, the proband and his younger sibling were both diagnosed with chronic kidney disease, and during the follow-up process, the proband progressed to ESRD by the time of submission; the proband’s mother was diagnosed with kidney disease at 31 years of age; the grandmother had been in good health and was only discovered to have proteinuria during a routine physical examination. Evidence indicates that the phenotypes of male family members are generally more severe than those of female members. This may be because women have two X chromosomes. To balance gene expression, one of the X chromosomes is randomly inactivated, which can occur as early as the embryonic period, and non-inactivated normal chromosomes can partially compensate, resulting in milder disease manifestations [23]. The phenotypic differences between the proband’s mother and grandmother may have been caused by inconsistencies in gene penetrance. Disease penetrance may depend on other genetic and environmental factors [24]. Blood tests showed that the glomerular filtration rate of the proband’s grandfather had decreased. He visited the hospital for kidney disease 10 years prior (the details are unknown), and later, he was in good health. We believe that his kidney damage was caused by reasons other than AS.

Currently, the Human Gene Mutation Database contains over 1000 versions of the *COL4A5* gene. More than 10% of these are splicing variations. In this study, an *in silico* assessment predicted that this variation may affect splicing. Splicing-site variations can cause truncated variation, which leads to premature codon termination, or non-truncated variation, which leads to exon jumping or intron retention. Only after the splicing variation is verified by in vitro experiments can its influence on the body be further evaluated. We used an ex vivo minigene splicing assay to confirm the abnormal splicing resulting from the mutation in *COL4A5*. The outcome indicated that the c.4298-8G > A mutation affected the regular splicing of *COL4A5* mRNA and produced a fresh acceptor site, leading to the retention of a 6-bp base (AAATAG) on the right side of intron 46 in COL4A5 transcripts. Consequently, two amino acids were inserted after the amino acid at position 1,432 of α5(IV). Moreover, the I-TASSER program was employed to predict the configuration of both wild-type and altered COL4A5 proteins and to construct a model. The hydrogen bond structure of amino acids 1432–1433 underwent changes both before and after the mutation. In addition, the electrostatic potential near the 1432_1433 mutation on the protein surface transitioned from electropositive to electronegative. These observations indicate that the c.4298-8G > A variant in *COL4A5* could potentially impact the structural abnormalities of the BM. Liang et al. discovered a comparable mutation, c.4298–20T > A, in the *COL4A5* gene. This mutation causes the retention of 18 bp in intron 46 of the *COL4A5* mRNA, resulting in the insertion of six amino acids after the amino acid at position 1432 in α5(IV). Molecular dynamics results showed that the c.4298–20T > A variant could affect the tail stability of the α345(IV) trimer as well as the stability of the head and middle of the α345(IV) trimer. Furthermore, the simulations suggest that the structure of the mutant α345(IV) trimer changes greatly and mutation has a great influence on their configuration [25]. In our study, although only two amino acids were inserted after the amino acid at position 1,432 in α5(IV), we suspect that this may also affect the stability of the α345(IV) trimer and the formation of a trimer configuration.

The genotype-phenotype correlation in male XLAS is relatively well established. Jais et al. reported that splicing mutations confer a 70% probability of ESRD development by 30 years of age [17]. Bekheirnia et al. reported that individuals with splice-site mutations experienced ESRD onset at an average age of 28 years old [11]. Horinouchi et al. reported that individuals with truncating mutations due to atypical splicing atypical developed ESRD at a median age of 20 years, whereas those with non-truncating mutations developed ESRD at a median age of 29 years [12], the study revealed a disparity in kidney prognosis between the two types of splicing variants. In our study, the c.4298–8G > A variant was a non-truncating mutation, and the proband’s two maternal uncles, who may have carried the *COL4A5* mutation, died of kidney disease at the ages of 24 and 10, respectively. The proband has also progressed to ESRD so far, and the younger brother also has kidney damage, the kidney damage caused by the *COL4A5* mutation is of serious phenotype in males. For the extrarenal phenotype, the proband and younger brother did not exhibit sensorineural hearing loss but only had a mild form of near-sightedness. In conclusion, the kidney phenotype caused by the splicing mutation c.4298-8G > A is considered serious.

However, the present study has some limitations. Due to insufficient renal biopsy tissue samples from the proband, we were unable to perform immunofluorescence testing. Although the pathogenicity of the novel *COL4A5* variant is explained by the clinical phenotype of the proband, his family history of kidney disease, pedigree analysis, bioinformatics predictions, and minigene assays, further functional studies are needed to determine the impact of the variant on *COL4A5* gene expression and function.

## Conclusions

We discovered a splicing variant, c.4298-8G > A, in the *COL4A5* gene in a patient with XLAS, and verified its splicing effect using a minigene assay. The mutation was assessed as likely pathogenic. This research broadens the range of mutations linked to the XLAS and enhances our understanding of the correlation between XLAS genotypes and phenotypes.

### Electronic supplementary material

Below is the link to the electronic supplementary material.

Supplementary Material 1

Supplementary Material 2

## Data Availability

The original contributions presented in the study are included in the article/Supplementary material. The raw datasets of participant generated during the current study are available in the NCBI Sequence Read Archive (SRA) database repository (https://www.ncbi.nlm.nih.gov/sra/?term=PRJNA1055030).

## Abbreviations

AS: Alport syndrome
XLAS: X-linked Alport syndrome
Hemi: hemizygote
Het: heterozygote
F: female
M: male
uPCR: urine protein to creatinine ratio
FSGS: focal segmental glomerulosclerosis
eGFR: estimated glomerular filtration rate
ESRD: end-stage renal disease
BM: basement membrane
ACMG: American College of Medical Genetics and Genomics
WES: whole-exome sequencing
