# Supplementary material for: A novel COL4A5 splicing mutation causes alport syndrome in a Chinese family
Source: BMC Med Genomics. 2024 Apr 26;17:108. doi: 10.1186/s12920-024-01878-8 (PMC11046743; doi:10.1186/s12920-024-01878-8)
Supplement: Supplementary file 1 — Supplementary Material 1 [file 12920_2024_1878_MOESM1_ESM.docx]

Table S1. Genes focused on and analyzed after high-throughput sequencing

| location | related genes |
| --- | --- |
| glomerular disease | ACSL4, ACTN4, ADA, AGPAT2, ALMS1, AMMECRI, ANLN, APOE, APOLI, ARHGDIA, BSCL2, BSND, CIQA, CIQB, CIQC, C3, C4A, CAVI, CAVIN1, CD2AP, CEP83, CFH, CFHR5, CFI, CLCN5, COL4A3, COL4A4, COL4A5, COPA, COQ2, COQ6, COQ8B, CRB2, CTLA4, DGKE, DNASE1L3, ELP1, EMP2, FAH, FAS, FH, FN1, FOXC2, G6PC, GATA3, GLA, HNF1B, HNF4A, IL10, IL23R, INF2, ITGA3, ITGB4, JAG1, KCNJ1, KIF1B, LAGE3, LAMB2, LMNA, LMNB2, LMX1B, LPIN2, MAGI2, MAX, MDH2, MEFV, MUCI, MYOIE, NARS2, NPHP3, NPHS1, NPHS2, NUP107, NUP133, NUP160, NUP205, NUP85, NUP93, OCRL.OSGEP, PAX2, PGM3, PLCE1, PLEC, PPARG, PRKCD, PTPN22, PTPRO, REN, RET, SCARB2, SDHA, SDHAF2, SDHB, SDHC, SDHD, SEC6IA1, SGPL1, SLCI2A1… 197genes. |
| nephritis | IRF5, MAPKBP1, MCEE, MMAA, MMAB, MMUT, MYH9, NEK8, NPHP1, NPHP3, NPHP4, PDCD1, PLG, PTPN22, SLEB3, SLEH1, SLEN1, SLEN2, SLEN3, STAT4, ANKS6, CASP10, CD151, CEP164, CEP83, COL4A3, COL4A4, COL4A5, CR2, CTLA4, DCDC2, DNASE1, DNASE1L3, FAN1, FCGR2A, FCGR2B, FN1, GLIS2, IFT172, INVS, TKCR, TLR5, TMEM67, TREX1, WDR19, XPNPEP3, 46 genes in total. |
| nephropathy | ITGA8, FGF20, RET, PAX2, UPK3A, GDNF, ROBO2, SOX17, TNXB, HNF1B, EYA1, SIX5SIX1, FRAS1, GRIP1, FREM2, HPSE2, LRIG2, GATA3, KAL1, FGFR1, SALL1, DIS3L2, GPC3, OFD1, REN, AGT, AGTR1, ACE, FGF18, FGF12, MAPK11, PDGFRA, AMH, DSTYK, BMP4, BMP7, CHDIL, WNT4, NPHP3, NEK8, VHL, PKD1, PKD2, PKHD1, MUC1, UMOD, NPHP1, NPHP4, IQCB1, CEP290, SDCCAG8, WDR19, RPGRIPIL, TMEM67, INVS. GLIS2, TTC21B, ZNF423, CEP164, ANKS6, INPP5E, TMEM216, AHI1, CC2D2A, TMEM237, CEP41, TMEM138, TCTN3, TMEM231, CSPP1, PDE6D, MKSI, TCTN2, B9D1, B9D2, IFT80, DYNC2HI, NEK1, WDR35, WDR60, IFTI40, IFTI72, WDR34, BBS1, BBS2, ARL6, BBS4, BBS5, MKKS, BBS7, TTC8, BBS9, BBS10, TRIM32, BBS12, WDPCP, LZTFL1, IFT122…264 genes. |
| urinary system | AAGAB, AASS, ABCAI2, ABCC2, ABCC6, ABCC8, ABCDI, ABCD4, ABCG5, ABCG8, ACAD9, ACADM, ACADS, ACADVL, ACE, ACP5, ACSF3, ACSL4, ACTAI, ACTB, ACTGI, ACTG2, ACTN4, ACVRLI, ADA, ADA2, ADAMTSI3, ADAMTS3, ADCYIO, ADGRG2, ADHIC, AFF4, AGA, AGK, AGPAT2, AGT, AGTRI, AGXT, AGXT2, AHII, AIP, AIRE, AKTI, AKT3, ALAD, ALDHI8AI, ALDH4AI, ALDOB, ALGI, ALGI2, ALG8, ALG9, ALK, ALMS1, ALOX12B, ALOXE3, ALPL, ALS2, ALX4, AMERI, AMMECRI, AMN, AMT, ANKLE2, ANKS6, ANLN, ANO5, ANOS1, ANTXR1, AP2S1, AP5Z1, APC, APC2, APOA1, APOB, APOE, APOL1, APRT, AQP2, AR, ARCNI, ARGI, ARHGDIA, ARIDIA, ARID1B, ARID2, ARL3, ARL6, ARMC5, ARNT2, ARSA, ARSB, ARX, ASL, ASPM, ASS1, ASXL1, ATAD3A, ATL1…1350 genes. |
