# Supplementary material for: A novel COL4A5 splicing mutation causes alport syndrome in a Chinese family
Source: BMC Med Genomics. 2024 Apr 26;17:108. doi: 10.1186/s12920-024-01878-8 (PMC11046743; doi:10.1186/s12920-024-01878-8)

Fig S1. The diagram of primers positions for specific alleles with both round 1 and 2.

Fig S2. The pcMINI-N vector map.

Fig S3. The agarose gel electrophoresis original image of RT-PCR.


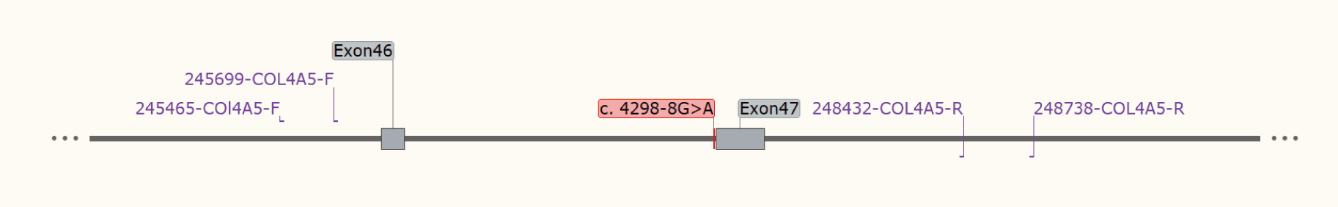


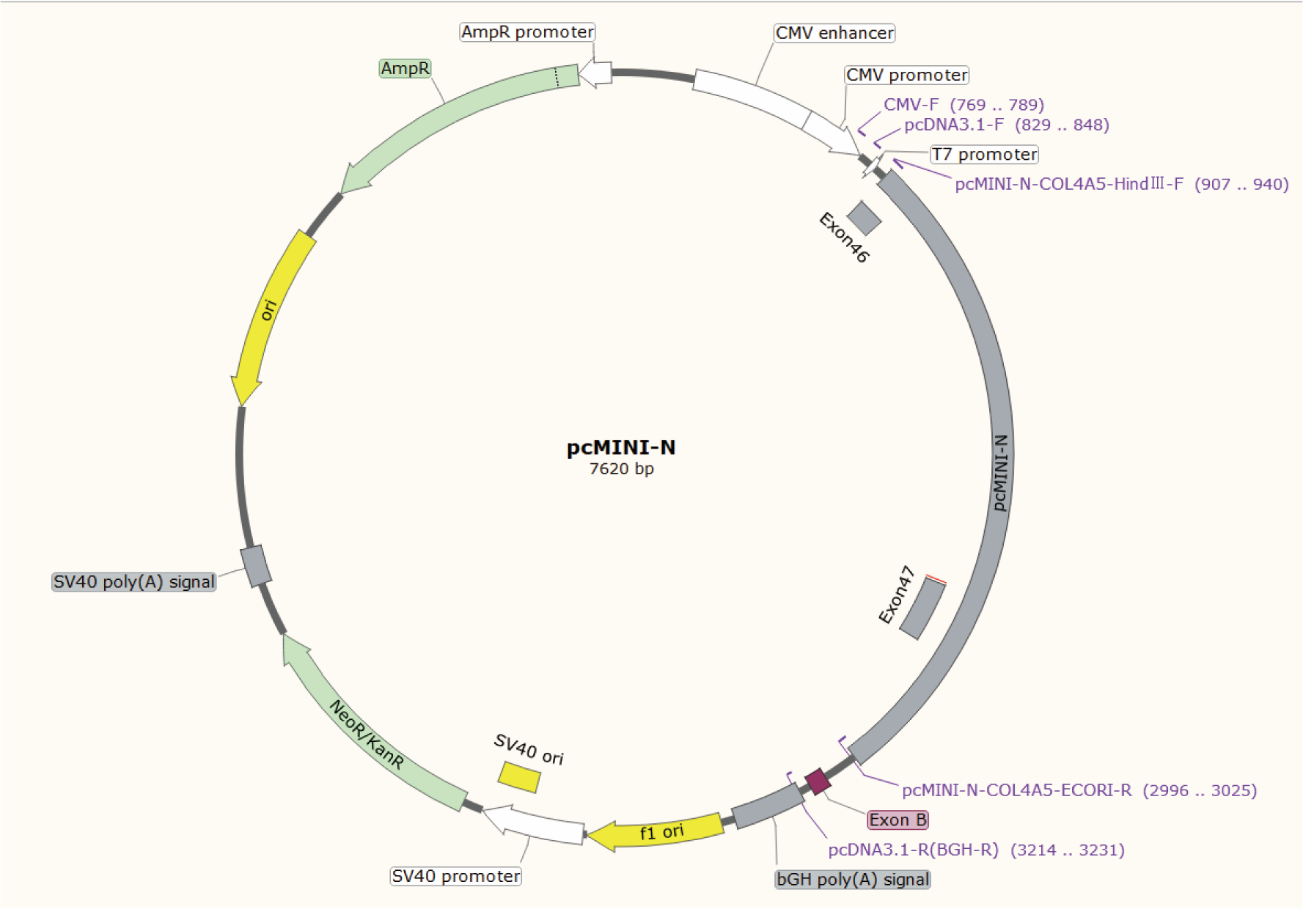


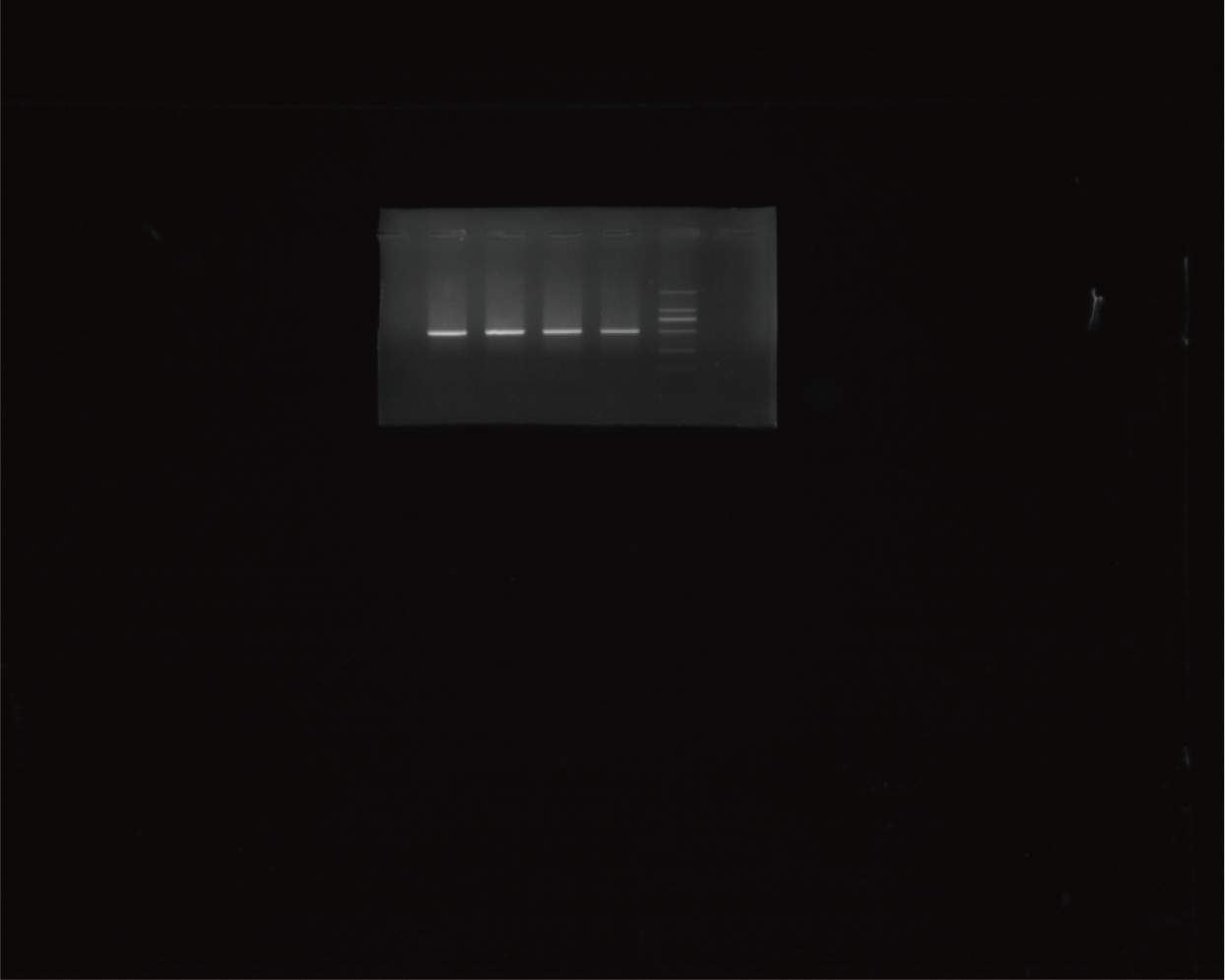

Supplement: Supplementary file 2 — Supplementary Material 2 [file 12920_2024_1878_MOESM2_ESM.docx]
